# Effects of Land Management Strategies on the Dispersal Pattern of a Beneficial Arthropod

**DOI:** 10.1371/journal.pone.0066208

**Published:** 2013-06-11

**Authors:** Chiara Marchi, Liselotte Wesley Andersen, Volker Loeschcke

**Affiliations:** 1 Department of Bioscience, Aarhus University, Aarhus, Denmark; 2 Department of Bioscience, Aarhus University, Rønde, Denmark; University of Helsinki, Finland

## Abstract

Several arthropods are known to be highly beneficial to agricultural production. Consequently it is of great relevance to study the importance of land management and land composition for the conservation of beneficial aphid-predator arthropod species in agricultural areas. Therefore our study focusing on the beneficial arthropod *Bembidion lampros* had two main purposes: I) identifying the physical barriers to the species' dispersal in the agricultural landscape, and II) assessing the effect of different land management strategies (i.e. use of pesticides and intensiveness) on the dispersal patterns. The study was conducted using genetic analysis (microsatellite markers) applied to samples from two agricultural areas (in Denmark) with different agricultural intensity. Land management effects on dispersal patterns were investigated with particular focus on: physical barriers, use of pesticide and intensity of cultivation.

The results showed that *Bembidion lampros* disperse preferably through hedges rather than fields, which act as physical barriers to gene flow. Moreover the results support the hypothesis that organic fields act as reservoirs for the re-colonization of conventional fields, but only when cultivation intensity is low. These results show the importance of non-cultivated areas and of low intensity organic managed areas within the agricultural landscape as corridors for dispersal (also for a species typically found within fields). Hence, the hypothesis that pesticide use cannot be used as the sole predictor of agriculture's effect on wild species is supported as land structure and agricultural intensity can be just as important.

## Introduction

A number of arthropods, typically living in agricultural areas, have been shown to be highly beneficial to agricultural production, because they act as predators on pest species [1]. These beneficial species are of major importance especially in relation to organic farming, and their abundance and dispersal pattern can be expected to be influenced by land management.

*Bembidion lampros*, a small polyphagus arthropod (of the Carabidae family) has been shown to be efficient as aphid pest controller in agricultural areas [1]–[3] and can also be used as indicator for insect abundance and diversity [4]. Effects of landscape structure and land use on *B. lampros* have been widely investigated, but focusing only on the species abundance rather than on the drivers of dispersal in the agricultural landscape.

The typical *B. lampros* life cycle is marked by an overwintering stage where individuals reside in hedges at the field margins and a dispersal stage in early spring where they move into the field [5]. The timing of dispersal is determined by air temperature and occurs in Denmark usually between early March and early April [5]. After reproducing in the field the individuals return in early autumn to the hedges to overwinter [6], [7]. *B. lampros* is univoltine [6] and short-lived but it lives mostly more than a year. It is one of the early spring-moving species [5], thus it has probably a relatively larger effect on aphid pest control compared to later-moving species [3]. While its life cycle has been thoroughly studied, the dispersal routes within the agricultural land are largely unknown. In particular it is not known whether *B. lampros* cross hedgerows, tend to return to the same hedgerow after reproduction or easily cross ploughed fields. Such knowledge however is crucial for understanding the population dynamics of this highly beneficial species [2]. Since this information is difficult to get through mark-recapture studies (given the very high population number and the small size of this species), the use of molecular genetic markers may help to elucidate this part of its ecology. Knowing which factors act as barrier to dispersal can be used to predict the probability of an area to receive immigrants and consequently be re-colonized in case of a sudden drop in abundance (e.g. after pesticides spraying). This knowledge could allow the implementation of management strategies that prevent populations going extinct or suffer severe bottlenecks (which could then lead to local extinction).

Hedgerow types, soil type, presence of pesticides and number of mechanical soil treatments have a clear effect on the abundance of *B. lampros* [8]–[10]. In particular many commonly used insecticides are shown to be detrimental to this species, both under laboratory and field conditions [11], [12]. Even pesticides legally used in organic fields in many European countries (but not in Denmark) have been proven to be harmful [9]. The use of pesticides in the field has led to a reduction in *B. lampros* abundance persisting up to four weeks after spraying; the following return to abundance levels preceding the spraying was ascribed to an effect of re-colonization from untreated nearby areas [13]. This might suggest that fields grown organically can act as genetic reservoirs for the re-colonization of conventional fields.

Several studies have shown the importance of cultivation intensity for the abundance and mobility of *B. lampros*, in particular of low yield cultivation with high weed abundance [14], [15]. Therefore the effect of agricultural intensiveness was included in our study by comparing two areas with very different agricultural intensity. The organic fields in the two areas also differed in time since conversion from conventional fields.

The main objectives of our study were :

- to analyze the population structure and gene flow pattern in the agricultural landscape and thereby identifying possibly dispersal barriers (with particular attention to fields, hedgerows and roads).
- to test the hypothesis that organically grown fields act as genetic reservoirs for conventional grown fields and the hypothesis that areas with low intensity agricultural land use have a higher genetic diversity, effective population size and comparatively higher gene flow.

## Materials and Methods

### Sampling design

We selected two agricultural areas with different intensity of cultivation composed of conventional and organic fields separated by tree-covered hedgerows. Sampling was performed for two consecutive years in early autumn (when individuals return to the hedgerows to overwinter) at sampling sites along the hedgerows.

**Figure 1:**
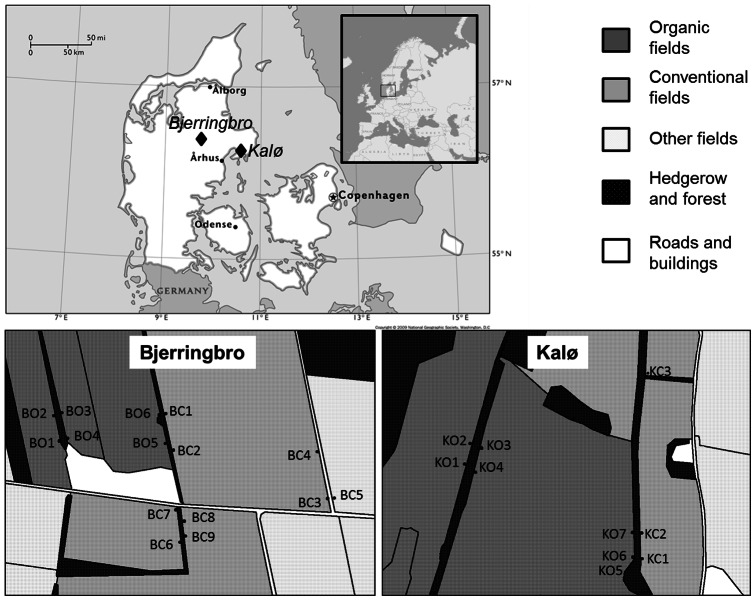
Map of sampling sites. The top map shows the location of the two areas used in this study. The maps at the bottom show the location and name of each sampling site for both areas (B = Bjerringbro, K = Kalø) and management types of the field (C for conventional or O for organic) and ordinal number.

Individuals were sampled along the hedgerows from two agricultural areas in Denmark, Kalø 56.363° North 9.700° East, and Bjerringbro 56.290° Nord 10.500° East. The two areas represented an intensive (Bjerringbro) and an extensive (Kalø) type of management: Bjerringbro area had a higher average amount of cultivated areas (66%) compared to the national level (61%), while the opposite was true for the Kalø area where only 52% of land is used for agriculture (Beate Strandberg, pers. comm.). In Kalø, the fields have been organically managed for many years (>15 years), longer than in Bjerringbro, and the weed cover in the fields in Kalø was also much higher (Beate Strandberg pers. comm.). The sampling scheme was designed to obtain samples from hedgerows dividing two organic or two conventional fields as well as from hedgerows dividing organic and conventional fields (on both sides). Regarding the pesticide regime the organic fields were never treated with pesticides/herbicides while these were routinely applied to conventional fields. Therefore conventional vs. organic classification defines pesticide regime. Each hedgerow presented four sampling sites 50 meters apart, two on both sites just opposite each other with a width of one meter. A total of 1140 samples were hand-picked during the two seasons (see Table 1 and Fig. 1 for details). Before DNA-extraction all individuals were identified to species morphologically.

**Table 1:** Sampling details.

	2008	2009	Total	Management
**Bjerringbro**				
BO1	28	0	28	Organic
BO2	28	0	28	Organic
BO3	24	17	41	Organic
BO4	29	27	56	Organic
BO5	34	28	62	Organic
BO6	20	22	42	Organic
BC1	30	29	59	Conventional
BC2	20	30	50	Conventional
BC3	23	9	32	Conventional
BC4	5	29	34	Conventional
BC5	0	25	25	Conventional
BC6	15	17	32	Conventional
BC7	9	7	16	Conventional
BC8	27	28	55	Conventional
BC9	26	26	52	Conventional
Total	318	294	612	
**Kalø**				
KO1	18	30	48	Organic
KO2	27	28	55	Organic
KO3	25	30	55	Organic
KO4	27	30	57	Organic
KO5	27	0	27	Organic
KO6	26	30	56	Organic
KO7	29	30	59	Organic
KC1	29	28	57	Conventional
KC2	30	30	60	Conventional
KC3	27	27	54	Conventional
Total	265	263	528	

Number of sampled individuals per sampling site, per year and in total (first to third column). Last column shows the type of management.

### DNA extraction and PCR details

Samples were frozen dry upon collection and stored at −20°C. DNA was extracted using a standard CTAB buffer and proteinase-K procedure [16], [17]. Two multiplex PCR runs were used to amplify 15 microsatellite markers developed specifically for this species (Marchi *et al*. unpublished) using Qiagen Multiplex enzyme following the manufactories instructions. First multiplex: BL8, BL11, BL19, BL22, BL28, BL28b, BL30, BL34; second multiplex: BL2, BL5, BL6, BL12, BL14, BL24, BL27). Thermal profile, for both multiplex runs, was: 95°C for 15 min; then 35 cycles of 30 s at 94°C, 45 s at 57°C and 60 s at 72°C, with a final extension at 60°C for 30 min. PCR products were analysed using an ABI 3730 automated sequencer and typed using GeneMapper version 4.1 (Applied Biosystems).

### Genetic diversity and effective population size analysis

Genetic diversity for each sampling site and each year was estimated as allelic richness with fstat [18] and expected heterozygosity with GenAlEx [19]. Tests for goodness of fit to Hardy-Weinberg expectations and linkage equilibrium were performed in fstat. Moreover effective population size (N_e_) was estimated with NeEstimator [20] for each sampling site for both years using “heterozygote excess method” [21] and Waples' [22] moments based approach. The differences in genetic diversity and N_e_ among conventional and organic sampling sites were tested using a Student t-test. The same tests were repeated, grouping the samples with regard to neighboring field's management as well (sampling site in conventional field with neighboring organic field versus sampling site in conventional field with neighboring conventional field and so on for all pairs).

### Genetic structure analysis

Population structure was evaluated using a Bayesian based cluster analysis implemented in geneland R package [23]. The analyses were performed for Kalø and Bjerringbro data using geographic coordinates as prior and k(max) = 10 (based on five separate runs each with 20,000/100,000 burn-in/sampling iterations).

The population structure was also estimated using pairwise multilocus *F*_st_ between sampling sites using arlequin v. 3.5 [24].

### Gene flow analysis

The effect of environmental variables on the pattern of gene flow between sampling sites were assessed using bimr [25]. bimr estimates recent gene flow to find the best explanatory factors for the recovered pattern among the given environmental distance matrices using a linear model fit with the gene flow matrix acting as dependent variable (a null model, including none of the specified factors, is always included in the analysis). The variables included in the analysis were: distance, hedgerow of belonging, field of belonging (hedgerow or field nearest to the sampling site), management type (conventional or organic) and road side (only for Bjerringbro). The inclusion of distance between sampling sites was used to account for possible effects of spatial autocorrelation. bimr analyses were run ten times for each area (using 1,000,000(burn in)/1,000,000(sampling) runs) and the gene flow values were obtained from the best run (which was selected on the basis of *D_assignment_* as suggested by Faubet et al. [26]; when more than one run presented the same *D_assignment_*, posterior probability and the visual analyses of likelihood plot for each run were used to determine the best run). BIMr results were analyzed also on the basis of the alpha value recovered for each factor (or combination of factor): “the sign and the magnitude of the alpha's tell us about the direction and the strength of the environmental factors” [25].

### Assignment test

Isolation of the clusters detected by Geneland was checked using a series of assignment tests conducted in GeneClass [27]. We performed four self-assignment tests using data from 2008 and 2009, separately, for each area. Then two assignment tests were performed assigning 2009 individuals using 2008 individuals as reference. For all six tests the individuals were divided according to the grouping recovered by the genetic structure analysis in Geneland. All tests were performed using Rannala and Mountain [28] method; 10,000 simulations with Paetkau et al. [29] method and a type I error rate *P*<0.01.

## Results

### Genetic diversity and effective population size analysis

No significant deviation from Hardy-Weinberg- or linkage equilibrium was found. The genetic diversity indexes were very similar across sampling sites with different management and across years. The Kalø area showed a significantly higher allelic richness compared to Bjerringbro area (Table S1). Effective population size did not show any difference across area, year or management type (Tables S1 and S2).

### Genetic structure analysis

**Figure 2:**
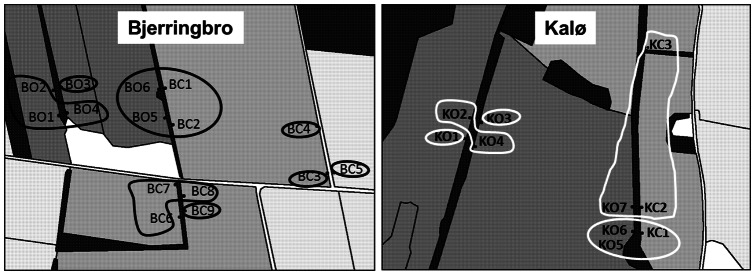
Genetic clusters recovered by GENELAND. The figure shows the genetic clusters for each area with each cluster being included in a solid line (black for Bjerringbro, white for Kalø).

Geneland analysis performed on samples from both years detected five clusters in Kalø and eight in Bjerringbro. In both cases no migrants were identified and samples from 2008 clustered together with the corresponding ones from 2009. All the sampling sites that did not constitute a cluster of their own were grouped accordingly to the nearest hedgerow (Fig. 2).

**Figure 3:**
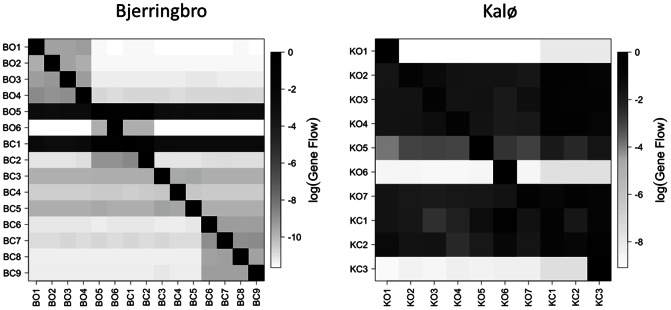
Pairwise *F*_st_ values. The pairwise *F*_st_ values for each pair of sampling sites are shown on a heatmap for both areas. Pairs that are significantly different, after sequential Bonferroni correction, are represented by a + sign on the heatmap.

The pairwise *F*_st_ analyses showed higher *F*_st_ values in Bjerringbro than in Kalø; despite the presence of significantly differentiated pairs, no isolated (group of) sampling sites were found (Fig. 3). However, in Kalø there were two more distinct groups: KO1-KO2-KO3-KO4 and KO7-KC1-KC2-KC3 (Fig. 3) which partly agreed with the results from Geneland (Fig. 2) as the sampling sites in the two groups belonged to the same hedgerow (confirming the effect of fields as genetic barriers).

### Gene flow analysis

BIMr analyses of Bjerringbro data converged in nine out of ten cases. “Hedgerow of belonging” was the most important factor in three out of nine runs. Further, in two single runs “road side” and “field of belonging”, respectively, were the most important explanatory factors. In other three runs “distance” (two runs) and “distance plus road side” (one run) were most important. When two factors were combined, “road side” and “field of belonging” were the least important ones (lowest absolute value of alpha). One run found the best fit to be the null model. The highest posterior probability and highest alpha were given to “hedgerow of belonging” alone. For the best run, the most important factor was “hedgerow of belonging” and the gene flow was higher for pairs of sampling sites belonging to the same hedgerow (see alpha values in Table 2).

**Table 2:** Results of gene flow analyses (Bjerringbro).

Most significant factors	Number of runs (total of 9 runs)	Posterior probability of the model	Alpha of the corresponding model
*Hedgerow*	3	*0.399*	*1.83*
		0.182	0.986
		0.352	1.51
Hedgerow + Road Side	1	0.242	Hedgerow:1.18
			Road Side:−0.9
Hedgerow + Field	1	0.191	Hedgerow: 0.996
			Field:−0.71
Distance	2	0.271	−1.58
		0.166	0.407
Distance + Road Side	1	0.103	Distance:−0.746
			Road Side:−0.477
Null model	1	0.155	1.35

For each of the 9 runs performed with BIMr the most significant factor(s), their posterior probability and the alpha value for that factor(s) are reported. The results from the best run (see Materials and methods for details) are shown in italics.

All ten runs regarding Kalø samples converged. In nine out of ten cases “management type” was the most important factor alone (four runs), together with “hedgerow of belonging” (4 runs) or together with” field of belonging” (1 run). In cases where two factors were present, “management type” was the most important one (highest absolute number of alpha). The best run found “management type” alone to be the most important factor (with a posterior probability of 0.582, see Table 3) and, as the correlation coefficient (alpha) was negative, the migration was lowest from conventional to organic sampling sites and highest from organic to conventional ones (see Materials and Methods for details).

**Table 3:** Results of gene flow analyses (Kalø).

Most significant factors	Number of runs (total of 10 runs)	Posterior probability of the model	Alpha of the corresponding model	
*Management*	4	*0.582*	*−1.58*	
		0.441	−1.23	
		0.368	−2.36	
		0.497	−2.06	
Hedgerow + Management	4		Hedgerow	Management
		0.490	0.497	−2.44
		0.310	0.571	−1.58
		0.359	0.625	−1.07
		0.267	0.732	−1.9
Field + Management	1	0.603	Field: −0.686	
			Management: −1.89	
Null model	1	0.300	0.339	

For each of the 10 runs performed with BIMr the most significant factor(s), their posterior probability and the alpha value for that factor(s) are reported. The results from the best run (see Materials and methods for details) are shown in italics.

In all cases, in both areas, belonging to the same hedgerow was found to increase gene flow (see alpha values for hedgerow in Tables 2 and 3).

### Assignment test

The self-assignment tests, performed on the samples from 2008 and 2009 for the two areas, were in good agreement with the population structure results as the percentage of mis-assigned individuals was generally low (Tables S3 and S4). The assignment test (2008 samples as reference for 2009) did not give clear cut results as most individuals could be assigned to more than one cluster. This was probably due to a lack of statistical power.

## Discussion

The main objectives of this study were: I) to determine the gene flow and the population structure patterns of *B. lampros* in agricultural areas with respect to landscape features (particularly: fields, hedgerows and roads) and II) to determine the effect of different land management strategies (specifically, pesticide use and agricultural intensiveness) on such patterns.

### Genetic diversity and effective population size analysis

Genetic diversity indexes and effective population sizes were similar across all sampling sites both within and across areas (with the exception of allelic richness which was much higher in Kalø). There was also no indication of an effect of management. The reason for this homogeneity might lie in the fact that the populations are not genetically isolated (as proven by the fact that the majority of pairs, in the *F*_st_ analysis, did not differ significantly from each other).

### Genetic structure analysis

The genetic structure analysis performed in Geneland showed that fields act as barriers to gene flow. The Geneland algorithm was chosen as it has been shown to be particularly good at finding genetic boundaries, especially when the population subdivision is recent [30]. Many sampling sites constituted a cluster of their own, while the ones that were grouped, always belonged to the same hedgerow. Management did not seem to affect the population structuring. It should also be noted that samples from the two separate years always clustered together showing that genetic structure is consistent through years.

**Figure 4:**
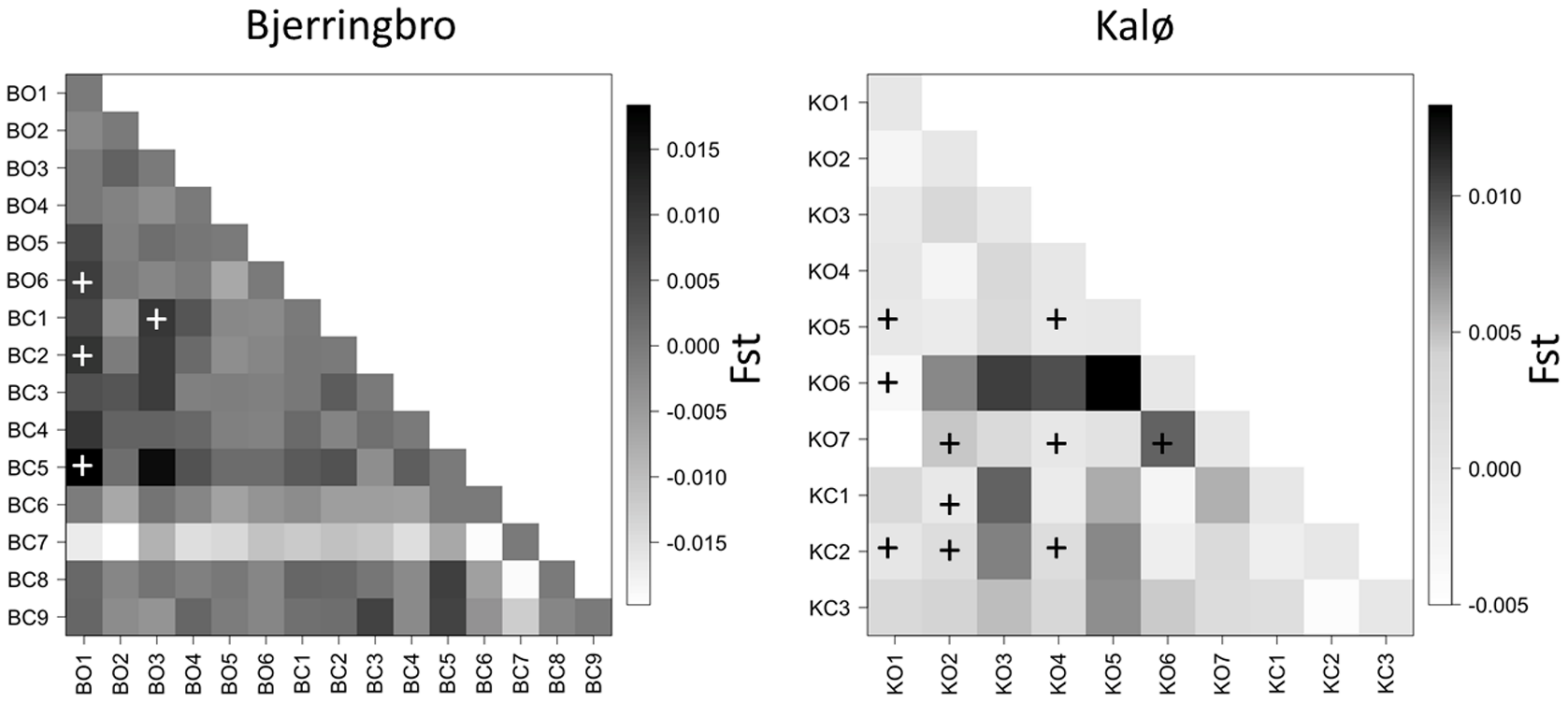
Gene flow values. The log_10_ of the gene flow values are shown on a heatmap for both areas. The populations of origin are shown on the vertical axis.

The patterns recovered by the *F*_st_ analysis were very different in the two areas (Fig. 4). The *F*_st_ values were higher in Bjerringbro than in Kalø; however, there were very few significantly different pairs in Bjerringbro compared to Kalø. In Kalø the *F*_st_ pattern is in good agreement with Geneland results as sampling sites lying on different field's sides are more differentiated than the ones sharing the same hedgerow. This results suggest a higher gene flow (lower *F*_st_ values), but an older isolation of sampling sites (more significantly different pairs) in Kalø compared to Bjerringbro. This difference might be due to a more consistent field structure in Kalø, where field management changed less over the years (Beate Strandberg, pers. comm.).

### Gene flow analysis

The analysis of gene flow confirmed the results of the *F*_st_ analysis, showing a much higher gene flow in the Kalø area compared to Bjerringbro (Fig. 3). The barrier effect of fields was also confirmed and hedgerows were shown to be the main dispersal path in both areas (i.e. the highest gene flow occurred between sampling sites belonging to the same hedgerow, Fig. 4). A study by Eyre et al. [8] has shown the importance of vegetation cover and crop type on the activity of *B. lampros* and, despite not dealing directly with dispersal patterns, activity in the field was found not to be correlated with activity in the fields' margin. However, in the Kalø area there was a very clear effect of management on the strength and direction of gene flow. In particular, the highest gene flow went from organic towards conventional fields (Fig. 4 right, last three columns). The fact that this result was not found for Bjerringbro area suggests that the factor that makes a difference is the degree of intensity of the management and that only a low intensity of cultivation allows organic fields to act as genetic reservoirs. The positive effect of low-intensity cultivation has been shown for many wild species such as: corn buntings [31] and farmland birds in general [32], European hare [33] and for a large number of arthropods [34]. Together with the previously mentioned studies, our work confirms, from a genetic point of view, the importance of low-intensity cultivations for the conservation of biodiversity. Moreover we show that, while organic fields can act as reservoirs for the re-colonization of conventional fields, the absence of pesticide use is not enough for a field to become a fauna-source for more managed areas. Therefore, our study discloses that (at least for this species) pesticide use and agricultural intensity are interdependent variables that should not be evaluated separately. It is however not possible to infer from these results the long-term effect of pesticide use and agricultural intensity due to the limited time-scale of the study (two years).

### Assignment tests

The results of self-assignment tests showed a good agreement with the genetic structure results as the number of mis-assigned individuals was generally low for each cluster. The results from Bjerringbro gene flow analyses showed that BO5 and BC1 are the most important source of emigrants; this is in accordance with the assignment tests from 2008 in which a lot of individuals from other clusters were assigned to the cluster containing these two populations. The results from Kalø for 2009 are also in agreement with the gene flow results as a lot of samples from other clusters were assigned to the clusters containing the conventional fields.

### Conclusions

All the presented results suggest that tree-covered hedgerows are the preferred dispersal route used by *B. lampros*. On the other hand, fields represent a barrier to gene flow, suggesting a tendency for individuals to return to the same hedgerow. However, low-intensity organic fields can be used for dispersal, probably due to a lower death rate caused by mechanical treatments and a higher concentration of aphids. This finding confirms the hypothesis of Huusela-Veistola [13] which suggested that the increase in *B. lampros* abundance following a sharp decrease after spraying was due to immigration from neighboring organic fields. Going beyond this, our results also confirm the importance of intensity of cultivation, and in particular of a low yield cultivation with high weed abundance [14], [15]. Future longer term and geographically broader studies might be able to determine with more accuracy the extent of the effect of each single variable.

Given the importance of *B. lampros* as a pest control agent, the results of this study can be important for the development of management strategies that maximize its beneficial effects [2]. Moreover, the fact that tree-covered hedgerows and low-intensity cultivation were found to be fundamental for the gene flow in agricultural areas strengthen the importance of including landscape features and cultivation intensity as parameters for the evaluation of land management strategies.

## Supporting Information

Table S1 **Genetic diversity indexes for each sampling site and year.** First column represent sampling site, (BO = Bjerringbro organic, BC = Bjerringbro conventional, KO = Kalø organic, KC = Kalø conventional) second column the year of sampling, third to fifth columns are the expected heterozigosity (H_e_) allelic richness (Ar) and effective population size respectively (N_e_) (see Materials and methods for details about their calculation).
(DOCX)

Table S2 **Effective population sizes for all sampling sites calculated with moment based method.** BO = Bjerringbro organic, BC = Bjerringbro conventional, KO = Kalø organic, KC = Kalø conventional.
(DOCX)

Table S3 **Results of self-assignment tests for Bjerringbro.** C1: BO1, BO2, BO4; C2: BO3; C3: BO5, BO6, BC1, BC2; C4: BC3; C5: BC4; C6: BC5; C7: BC6, BC7, BC8; C8: BC9.
(DOCX)

Table S4 **Results of self-assignment tests for Kalø.** C1: KO; C2: KO2, BO4; C3: KO3; C4: KO5, KO6, KC1; C5: KO7, KC2, KC3.
(DOCX)
