# Supplementary material for: Effects of Land Management Strategies on the Dispersal Pattern of a Beneficial Arthropod
Source: PLoS One. 2013 Jun 11;8(6):e66208. doi: 10.1371/journal.pone.0066208 (PMC3679026; doi:10.1371/journal.pone.0066208)
Supplement: Table S1 — Genetic diversity indexes for each sampling site and year. First column represent sampling site, (BO = Bjerringbro organic, BC = Bjerringbro conventional, KO = Kalø organic, KC = Kalø conventional) second column the year of sampling, third to fifth columns are the expected heterozigosity (He) allelic richness (Ar) and effective population size respectively (Ne) (see Materials and methods for details about their calculation). (DOCX) [file pone.0066208.s001.docx]

|  | **Year** | **H_e_** | **Ar** | **N_e_** |
| --- | --- | --- | --- | --- |
| **BO1** | 2008 | 0.618 | 1.664 | 2.1 |
| **BO2** | 2008 | 0.610 | 1.626 | 4.8 |
| **BO3** | 2008 | 0.597 | 1.611 | NA |
| **BO4** | 2008 | 0.627 | 1.641 | 20 |
| **BO5** | 2008 | 0.631 | 1.642 | NA |
| **BO6** | 2008 | 0.651 | 1.668 | 10.1 |
| **BC1** | 2008 | 0.680 | 1.693 | 285.9 |
| **BC2** | 2008 | 0.660 | 1.678 | 9.6 |
| **BC3** | 2008 | 0.667 | 1.685 | 8.4 |
| **BC4** | 2008 | 0.626 | 1.734 | 6.8 |
| **BC6** | 2008 | 0.620 | 1.643 | 5.9 |
| **BC7** | 2008 | 0.667 | 1.712 | 25.1 |
| **BC8** | 2008 | 0.656 | 1.670 | 9.1 |
| **BC9** | 2008 | 0.631 | 1.662 | 2 |
| **BO3** | 2009 | 0.656 | 4.646 | 5.3 |
| **BO4** | 2009 | 0.655 | 4.664 | NA |
| **BO5** | 2009 | 0.635 | 4.477 | NA |
| **BO6** | 2009 | 0.639 | 4.516 | 18.4 |
| **BC1** | 2009 | 0.634 | 4.490 | 17.4 |
| **BC2** | 2009 | 0.623 | 4.328 | 17.6 |
| **BC3** | 2009 | 0.617 | 4.619 | 7.7 |
| **BC4** | 2009 | 0.645 | 4.487 | 102.3 |
| **BC5** | 2009 | 0.636 | 4.367 | 14.5 |
| **BC6** | 2009 | 0.610 | 4.275 | 22.6 |
| **BC7** | 2009 | 0.544 | NA | 22.6 |
| **BC8** | 2009 | 0.619 | 4.448 | 118.3 |
| **BC9** | 2009 | 0.645 | 4.393 | 13.3 |
| **KO1** | 2008 | 0.662 | 2.697 | 4.5 |
| **KO2** | 2008 | 0.647 | 2.578 | 66.7 |
| **KO3** | 2008 | 0.653 | 2.601 | 7.7 |
| **KO4** | 2008 | 0.637 | 2.542 | 36.4 |
| **KO5** | 2008 | 0.658 | 2.596 | 6.1 |
| **KO6** | 2008 | 0.648 | 2.580 | 3.9 |
| **KO7** | 2008 | 0.633 | 2.517 | 9.9 |
| **KC1** | 2008 | 0.604 | 2.443 | 11.9 |
| **KC2** | 2008 | 0.633 | 2.521 | 12.1 |
| **KC3** | 2008 | 0.641 | 2.519 | 15.8 |
| **KO1** | 2009 | 0.644 | 5.062 | 17.3 |
| **KO2** | 2009 | 0.644 | 5.082 | 6.4 |
| **KO3** | 2009 | 0.641 | 5.005 | 5.8 |
| **KO4** | 2009 | 0.643 | 4.918 | 4 |
| **KO5** | 2009 | 0.686 | 5.457 | NA |
| **KO7** | 2009 | 0.635 | 5.138 | NA |
| **KC1** | 2009 | 0.636 | 5.017 | 43.2 |
| **KC2** | 2009 | 0.613 | 4.802 | NA |
| **KC3** | 2009 | 0.594 | 4.634 | 15.1 |
