# Supplementary material for: Effects of Land Management Strategies on the Dispersal Pattern of a Beneficial Arthropod
Source: PLoS One. 2013 Jun 11;8(6):e66208. doi: 10.1371/journal.pone.0066208 (PMC3679026; doi:10.1371/journal.pone.0066208)
Supplement: Table S2 — Effective population sizes for all sampling sites calculated with moment based method. BO = Bjerringbro organic, BC = Bjerringbro conventional, KO = Kalø organic, KC = Kalø conventional. (DOCX) [file pone.0066208.s002.docx]

|  | Moment based N_e_ | 95% CI |
| --- | --- | --- |
| BO1 | NA | NA |
| BO2 | NA | NA |
| BO3 | 5.3 | (2.2, 5.3) |
| BO4 | 3.2 | (2.3, 4.4) |
| BO5 | 2.6 | (1.9, 3.6) |
| BO6 | 4.2 | (2.8, 6.3) |
| BC1 | 3.5 | (2.8, 4.8) |
| BC2 | 3.5 | (2.5, 5.1) |
| BC3 | 3.9 | (2.4, 6.4) |
| BC4 | 5.9 | (2.9, 17.6) |
| BC5 | NA | NA |
| BC6 | NA | NA |
| BC7 | NA | NA |
| BC8 | 3.2 | (2.3, 4.5) |
| BC9 | 6.6 | (4.0, 11.6) |
| KO1 | 3.9 | (2.5, 6.1) |
| KO2 | 2.7 | (1.9, 3.7) |
| KO3 | 5.6 | (3.7, 8.5) |
| KO4 | 3.2 | (2.2, 4.5) |
| KO5 | 3.4 | (2.4, 4.7) |
| KO6 | NA | NA |
| KO7 | 3.2 | (2.2, 4.4) |
| KC1 | 4.5 | (3.0, 6.5) |
| KC2 | 4.0 | (2.8, 5.6) |
| KC3 | 2.4 | (1.7, 3.3) |
