# Supplementary material for: Effects of Land Management Strategies on the Dispersal Pattern of a Beneficial Arthropod
Source: PLoS One. 2013 Jun 11;8(6):e66208. doi: 10.1371/journal.pone.0066208 (PMC3679026; doi:10.1371/journal.pone.0066208)
Supplement: Table S3 — Results of self-assignment tests for Bjerringbro. C1: BO1, BO2, BO4; C2: BO3; C3: BO5, BO6, BC1, BC2; C4: BC3; C5: BC4; C6: BC5; C7: BC6, BC7, BC8; C8: BC9. (DOCX) [file pone.0066208.s003.docx]

| SA08 | **C1** | **C2** | **C3** | **C4** | **C5** | **C6** | **C7** | **C8** |
| --- | --- | --- | --- | --- | --- | --- | --- | --- |
| **C1** | 0.41 | 0.04 | 0.21 | 0.05 | 0.06 | NA | 0.19 | 0.05 |
| **C2** | 0.00 | 0.88 | 0.04 | 0.00 | 0.04 | NA | 0.04 | 0.00 |
| **C3** | 0.00 | 0.00 | 0.87 | 0.04 | 0.05 | NA | 0.03 | 0.00 |
| **C4** | 0.00 | 0.00 | 0.13 | 0.61 | 0.13 | NA | 0.09 | 0.04 |
| **C5** | 0.00 | 0.00 | 0.00 | 0.00 | 1.00 | NA | 0.00 | 0.00 |
| **C6** | NA | NA | NA | NA | NA | NA | NA | NA |
| **C7** | 0.00 | 0.00 | 0.10 | 0.12 | 0.14 | NA | 0.65 | 0.00 |
| **C8** | 0.12 | 0.00 | 0.15 | 0.00 | 0.04 | NA | 0.04 | 0.62 |
| SA09 | **C1** | **C2** | **C3** | **C4** | **C5** | **C6** | **C7** | **C8** |
| **C1** | 1.00 | 0.00 | 0.00 | 0.00 | 0.00 | 0.00 | 0.00 | 0.00 |
| **C2** | 0.18 | 0.82 | 0.00 | 0.00 | 0.00 | 0.00 | 0.00 | 0.00 |
| **C3** | 0.17 | 0.06 | 0.63 | 0.06 | 0.01 | 0.03 | 0.05 | 0.00 |
| **C4** | 0.11 | 0.00 | 0.00 | 0.89 | 0.00 | 0.00 | 0.00 | 0.00 |
| **C5** | 0.10 | 0.00 | 0.03 | 0.00 | 0.86 | 0.00 | 0.00 | 0.00 |
| **C6** | 0.12 | 0.00 | 0.04 | 0.04 | 0.00 | 0.80 | 0.00 | 0.00 |
| **C7** | 0.10 | 0.02 | 0.06 | 0.00 | 0.00 | 0.02 | 0.79 | 0.00 |
| **C8** | 0.15 | 0.04 | 0.00 | 0.00 | 0.00 | 0.00 | 0.08 | 0.69 |
