# Supplementary material for: Effects of Land Management Strategies on the Dispersal Pattern of a Beneficial Arthropod
Source: PLoS One. 2013 Jun 11;8(6):e66208. doi: 10.1371/journal.pone.0066208 (PMC3679026; doi:10.1371/journal.pone.0066208)
Supplement: Table S4 — Results of self-assignment tests for Kalø. C1: KO; C2: KO2, BO4; C3: KO3; C4: KO5, KO6, KC1; C5: KO7, KC2, KC3. (DOCX) [file pone.0066208.s004.docx]

| 2008 | **C1** | **C2** | **C3** | **C4** | **C5** |
| --- | --- | --- | --- | --- | --- |
| **C1** | 0.94 | 0.00 | 0.00 | 0.06 | 0.00 |
| **C2** | 0.00 | 0.85 | 0.00 | 0.00 | 0.06 |
| **C3** | 0.00 | 0.08 | 0.84 | 0.00 | 0.08 |
| **C4** | 0.01 | 0.04 | 0.00 | 0.79 | 0.16 |
| **C5** | 0.00 | 0.03 | 0.03 | 0.06 | 0.86 |
| 2009 | **C1** | **C2** | **C3** | **C4** | **C5** |
| **C1** | 0.63 | 0.00 | 0.00 | 0.23 | 0.13 |
| **C2** | 0.07 | 0.40 | 0.05 | 0.41 | 0.05 |
| **C3** | 0.03 | 0.07 | 0.60 | 0.23 | 0.03 |
| **C4** | 0.00 | 0.00 | 0.00 | 1.00 | 0.00 |
| **C5** | 0.10 | 0.01 | 0.00 | 0.28 | 0.60 |
